# Single-dose DMT reverses anhedonia and cognitive deficits via restoration of neurogenesis in a stress-induced depression model

**DOI:** 10.1038/s41398-026-03852-7

**Published:** 2026-01-29

**Authors:** Rafael V. Lima da Cruz, Rêmullo B. G. de Miranda Costa, Gabriel M. de Queiroz, Tijana Stojanovic, Thiago C. Moulin, Richardson N. Leão

**Affiliations:** 1 Neurodynamics Lab, Brain Institute (ICe) Universidade Federal do Rio Grande do Norte, Natal, Brazil; 2 Neurogenetics Lab, Brain Institute (ICe) Universidade Federal do Rio Grande do Norte, Natal, Brazil; 3 Translational Physiology and Pharmacology Program, Karolinska Institutet, Stockholm, Sweden; 4 Department of Pharmaceutical Biosciences, Uppsala University, Uppsala, Sweden; 5 Department of Surgical Sciences, Uppsala University, Uppsala, Sweden

**Keywords:** Depression, Molecular neuroscience

## Abstract

Major depressive disorder (MDD) remains a leading cause of disability worldwide, with current treatments limited by delayed onset and low efficacy. The serotonergic psychedelic N,N-dimethyltryptamine (DMT) has shown rapid antidepressant effects in early clinical studies, yet its mechanisms and efficacy remain poorly characterized in established models of depression. Here, we evaluated the effects of a single dose of DMT (30 mg/kg, i.p.) in male mice exposed to the Chronic Unpredictable Mild Stress (UCMS) paradigm, a robust mouse model recapitulating key features of MDD, including anhedonia and cognitive impairment. DMT administered after UCMS reversed depressive-like behavior and restored cognitive performance, outperforming chronic fluoxetine across most domains. When administered during the stress period, DMT mitigated anhedonic responses but did not rescue cognitive deficits, suggesting a long-lasting domain-specific efficacy. Exploratory assessments in anesthetized animals showed that DMT’s behavioral and cellular benefits persisted under isoflurane, though the role of the psychedelic experience remains uncertain due to potential confounding effects of isoflurane not controlled for in our design. Histological analyses revealed that all DMT regimes significantly increased adult-born granule cell (abGC) integration and reduced the number of ectopically abnormally integrated abGCs. Together, our findings highlight the robust and multifaceted effects of DMT on behavior and neurogenesis, positioning it as a promising candidate for rapid-acting antidepressant strategies that target structural circuit repair.

## Introduction

Major depressive disorder (MDD) is among the most prevalent and debilitating mental illnesses worldwide. According to recent estimates, 280 million people, including 5% of all adults, experience depression [1]. This disorder is strongly associated with reduced quality of life, cognitive abilities, and work productivity [2]. While conventional antidepressants such as selective serotonin reuptake inhibitors (SSRIs) have demonstrated therapeutic efficacy, their clinical limitations are substantial. These include a delayed onset of therapeutic action, often taking several weeks, and a high rate of treatment resistance, with approximately 30% of patients showing inadequate responses despite optimized interventions [3]. This has created a critical need for the development of more effective and fast-acting treatment strategies.

In recent years, accumulating evidence has highlighted the role of adult hippocampal neurogenesis in the pathophysiology and treatment of depression. The dentate gyrus (DG), a subregion of the hippocampus, continually integrates adult-born granule cells (abGCs) into its circuitry, a process essential for cognitive flexibility and mood regulation [4, 5]. Activation of abGCs has been shown to be sufficient to alleviate depression-like phenotypes, reversing the effects of chronic stress [6]. Additionally, most effective antidepressant treatments associated with improved behavioral outcomes enhance neurogenesis, including SSRIs [7, 8], lithium [9, 10], ketamine [11, 12], and even non-pharmacological interventions such as electroconvulsive shock [13, 14]. These findings support the neurogenic hypothesis of depression, which posits that disrupted neurogenesis contributes to depressive symptomatology and, thus, targeting neurogenic factors may yield therapeutic benefits [4, 15].

Among emerging therapeutic options, serotonergic psychedelics have garnered significant attention for their rapid and sustained antidepressant effects. Compounds such as psilocybin, LSD, and mescaline, classified as classical psychedelics due to their potent 5-HT2A receptor agonism, have demonstrated the ability to rapidly alleviate depressive symptoms, often within hours after a single administration [16–19]. These compounds have been shown to enhance neuroplasticity and promote various aspects of neurogenesis, including the proliferation, migration, and differentiation of neurons [20]. Specifically, activation of the 5-HT2A receptor promotes both precursor proliferation and neuronal differentiation in the adult DG, supporting its role in facilitating neuroplastic adaptations relevant to antidepressant efficacy [21, 22]. Other serotonergic targets, like 5-HT1A and 5-HT2C, may also mediate neurogenic effects of psychedelics. The 5-HT1A receptor, in particular, has been shown to regulate progenitor proliferation and neuronal survival [23, 24]. In parallel, 5-HT2C receptors influence the survival and differentiation of late-stage progenitors, exerting additional control over neurogenic output in the DG [21]. These complementary actions suggest that the therapeutic effects of psychedelics may involve the coordinated engagement of multiple receptor pathways, which act across different stages of neurogenesis to reshape hippocampal circuits [22].

Supporting this view, psilocybin has demonstrated robust and prolonged antidepressant efficacy in clinical trials, with symptom improvements persisting for up to 12 months in some patients and a favorable safety profile [25–29]. Moreover, ‘N,N-dimethyltryptamine (DMT) is another promising antidepressant compound. It is a key psychoactive ingredient in *ayahuasca*, a traditional Amazonian brew used ceremonially for centuries [30]. The long-standing, widespread use of DMT across various populations for centuries bolsters the evidence for its potential safety profile in the context of antidepressant applications with remarkable tolerability [31]. Like psilocybin, DMT primarily targets the 5-HT2A receptor but also binds to 5-HT1A and 5-HT2C receptors. Furthermore, the DMT molecule naturally occurs in various organisms, including humans [32]. When administered intravenously or inhaled, DMT bypasses rapid first-pass metabolism and induces intense but short-lived psychedelic experiences, characterized by profound alterations in perception, affect, and cognition [33, 34].

Despite decades of observational and pharmacological research, the clinical potential of DMT has only recently begun to be systematically evaluated [35]. For example, Osorio et al. [36] reported rapid reductions in depression scores following a single dose of *ayahuasca* in treatment-resistant patients, with effects sustained over several weeks. Palhano-Fontes et al. [37] conducted a randomized placebo-controlled trial confirming that *ayahuasca* produced a significant and rapid antidepressant effect compared to placebo. In a longitudinal study, Jiménez-Garrido et al. [38] reported that over 80% of participants who met the diagnostic criteria for a psychiatric disorder exhibited sustained clinical improvements six months after *ayahuasca* use. More recently, studies administering pure DMT have begun to emerge. For instance, A recent open-label trial showed that inhaled DMT produced rapid and sustained antidepressant effects in patients with treatment-resistant depression [39]. The treatment was safe, well-tolerated, and led to a significant reduction in depressive symptoms within 7 days, with response and remission rates of 85.7 and 57.1%, respectively. Effects lasted up to three months, and suicidal ideation dropped significantly, highlighting DMT’s potential as a fast-acting and scalable therapeutic option. Moreover, in an exploratory open-label trial for dose-related effects, intravenous DMT administration to MDD treatment-resistant individuals produced a rapid and significant decrease in depression scores, with mostly mild adverse events reported [40].

Beyond psychiatric outcomes, recent preclinical studies have elucidated DMT’s ability to modulate neural plasticity. Rodent models have shown that both high-dose and microdosed DMT facilitate fear-extinction learning and produce antidepressant-like effects [41, 42]. Likewise, 5-MeO-DMT, a structural analog of DMT with similar psychedelic properties, has shown robust anxiolytic effects in rodents subjected to stress [43, 44]. At the cellular level, DMT enhances neurite outgrowth, synaptic density, and dendritic spine formation in cortical neurons, comparable to the effects of ketamine [45]. Moreover, our group has previously shown that a single 5-MeO-DMT administration increases adult hippocampal neurogenesis and alters the electrophysiological properties of newborn neurons in the DG, conferring heightened excitability and synaptic plasticity to abGCs [46]. These neuroplastic changes are further supported by molecular findings showing upregulation of brain-derived neurotrophic factor (BDNF) and activation of sigma-1 receptors, both implicated in mood regulation and neurogenesis [20, 22, 47].

Despite promising clinical and preclinical findings, key questions remain regarding the therapeutic potential of DMT in depression. Notably, no study to date has systematically examined the effects of pure DMT in a validated preclinical model of depression-like behavior. Here, we address this critical gap by using the Unpredictable Chronic Mild Stress (UCMS) paradigm in mice, a well-established model that captures core features of MDD, particularly anhedonia [48]. Furthermore, while previous studies have shown that DMT can reverse depressive symptoms, it remains unknown whether it can remain effective when administered during periods of chronic stress. To investigate this, we administered a single dose of DMT not only after, but also during the UCMS protocol. A final unresolved issue concerns the role of subjective psychedelic experiences in mediating therapeutic effects. While some authors argue that the hallucinogenic conscious experience is necessary for enduring clinical benefits [49], others suggest that psychedelics act through downstream molecular and synaptic mechanisms that might be separable from conscious perception [50]. Here, we sought to explore this question and provide initial evidence by administering DMT under isoflurane, a widely used anesthetic in animal research, to examine potential interactions between anesthesia and DMT’s behavioral and cellular effects. We also performed histological analysis of abGCs, which were temporally labeled using the DCXCreERT2::TdTOM^lox/lox^ system. Together, our findings offer novel phenotypic and mechanistic insights into how altered neurogenesis and abGC circuit integration contribute to the pathophysiology of depression and its reversal by psychedelics such as DMT.

## Materials and methods

### Drugs

DMT (N,N-Dimethyltryptamine) was extracted and purified in-house from *Mimosa tenuiflora* root bark, collected in the municipality of Quixadá, Ceará, Brazil. The bark was dried at 50 °C, ground into a fine powder, and stored in airtight containers until use. The DMT free base was isolated from this powdered root bark. The material was suspended in 0.5 M sodium hydroxide (NaOH) at a ratio of 0.1 g of plant material per mL of alkaline solution. The mixture was magnetically stirred at room temperature (25 °C), protected from light, for 24 h. The extract was separated by vacuum filtration, and the plant residue was discarded. The aqueous phase was extracted twice with hot hexane (45 °C) using a 2:1 (v/v) solvent-to-aqueous ratio. The organic layers were combined, concentrated under reduced pressure to approximately 10 mL, and stored at –4 °C. After 24 h, light yellow crystals formed, which were filtered and recrystallized twice from hexane. The resulting DMT crystals were analyzed by gas chromatography-mass spectrometry (GC-MS). The average purity was 99.0%, with the lowest purity lot measuring 98.4%. The compound was stored in dimethyl sulfoxide (DMSO) at −20 °C and diluted fresh in saline (1:9, DMSO:saline) on the day of treatment to reach 30 mg/kg dose.

Fluoxetine was procured in pure form from a local compounding pharmacy (Ao Pharmacêutico, Natal, Brazil) and diluted in saline to a 20 mg/kg dose prepared fresh twice a week. Tamoxifen (Sigma-Aldrich, T5648) was prepared in autoclaved sesame oil (Sigma-Aldrich, S3547) in 100 µg/kg and stored at 4 °C for no longer than five days.

Injection volumes for all treatments, including fluoxetine and tamoxifen, were individually adjusted based on each animal’s body weight, with a maximum volume not exceeding 10 μL per gram. For instance, a 30 g mouse received up to 300 μL per injection. Injections were predominantly administered during the morning hours, unless otherwise specified by the experimental protocol. Due to the large number of procedures and the need to coordinate multiple treatment groups within a constrained schedule, occasional deviations were required to ensure timely delivery of all planned interventions.

### Animals and treatment

**Fig. 1:**
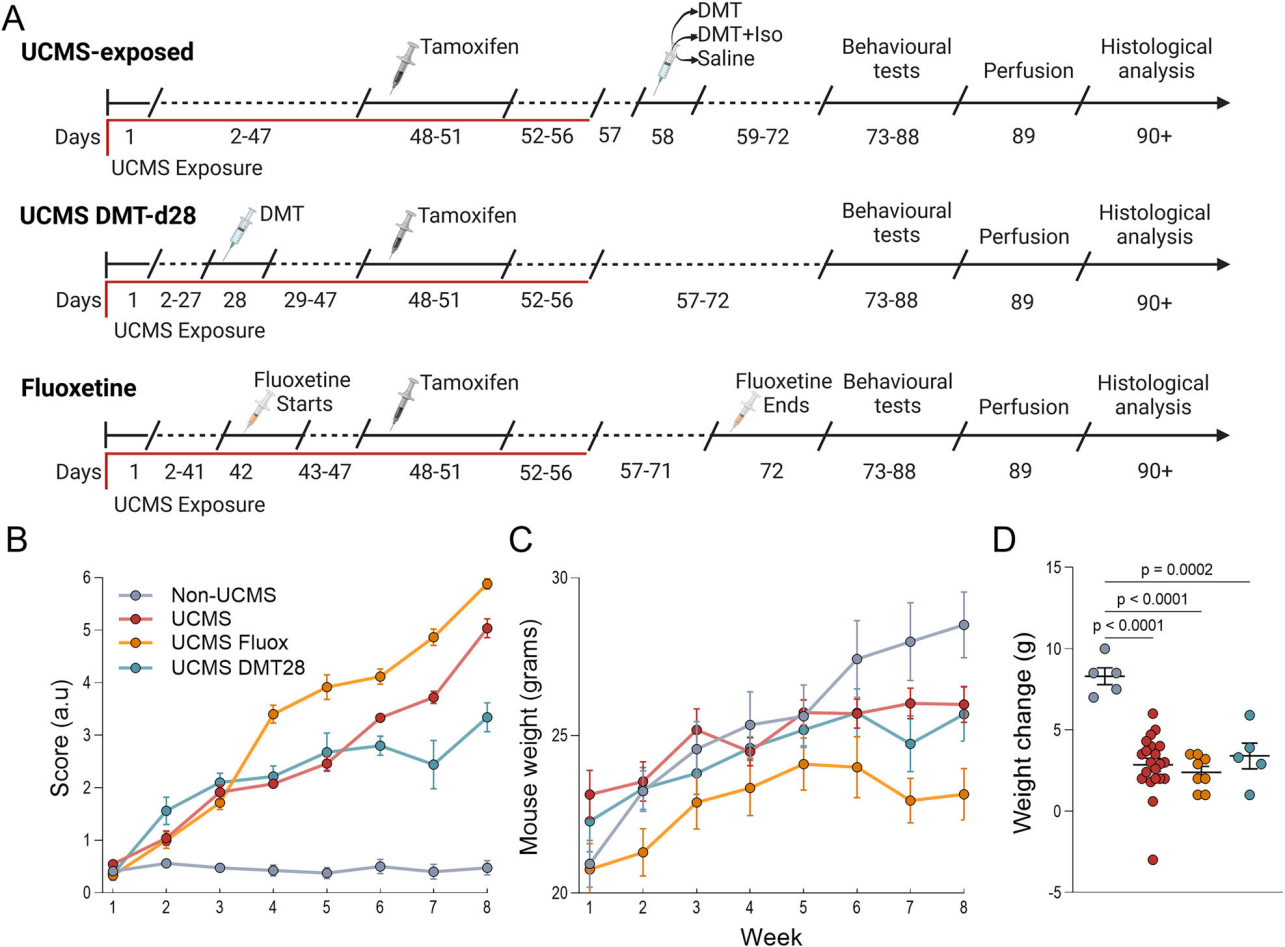
Experimental Design and Outcome Measures. **A** Timeline of experimental groups. The upper row represents UCMS-exposed animals, which underwent the UCMS paradigm from days 1–47, followed by a single injection of either Saline, DMT, or DMT+Iso on day 58. The middle row represents the UCMS DMT-d28 group, which followed the same UCMS exposure timeline but received a single DMT treatment on day 28. The bottom row represents the Fluoxetine group, in which animals started chronic fluoxetine treatment on day 42, continuing through day 72, overlapping with UCMS exposure. All groups received tamoxifen (days 48–51) and underwent behavioral testing (days 73–88), perfusion (day 89), and histological analysis (days 90 + ). **B** Longitudinal coat state scoring, where higher scores indicate worsening fur condition. **C** Longitudinal body weight assessment. **D** Comparison of final weight gain (week 8 – week 1) across experimental groups. Analyses in panels were conducted using either two-way ANOVA (in B and C) or one-way ANOVA followed by Tukey’s post-hoc test (in D); adjusted p-values are shown in the figure. Sample sizes: Non-UCMS (n = 5), UCMS (n = 21), UCMS Fluoxetine (n = 8), and UCMS DMT-d28 (n = 5).

A total of 48 male DCXCreERT2: :TdTOM^lox/lox^ mice with a C57BL/6 genetic background, aged postnatal day 45–70, were used in this study. The decision to use only male animals was based on prior evidence indicating that the UCMS protocol does not reliably induce robust behavioral phenotypes in female mice of the C57BL/6 strain, which matches the genetic background of our transgenic line [51]. The baseline average body weight of the animals across groups was 21.77 g. Mice were obtained from a local biotherium and randomly assigned to six experimental groups, while ensuring that littermates were distributed across different treatment conditions to minimize potential confounds related to shared housing or genetic background. This approach preserved the benefits of randomization while controlling for litter and cage effects. All mice were housed under standard conditions, including a 12 h light/dark cycle, with food and water available ad libitum. During the Sucrose Preference Test (SPT) and Delayed Non-Matching to Place (DNMP) tasks, housing and environmental parameters such as food, water, and lighting were adjusted to align with the respective testing protocols. Experiments were conducted in six cohorts of eight animals each, with each cohort including at least one representative from each treatment group to maintain consistency across experimental conditions. The experimental design is illustrated in Fig. 1A and Supplementary Fig. 1.

Mice in all DMT-treated groups received a single intraperitoneal injection of 30 mg/kg DMT, administered in a volume corresponding to animal weight × 10 µl (e.g., 200 µl for a 20 g mouse), and were then returned to their home cages with ad libitum access to food and water. Dose selection was guided by pharmacological data from Blair et al. [52], who reported hallucinogen-like substitution of DMT for LSD in a drug discrimination paradigm. From these data, we derived a dose-response curve using probit regression (Probit = 1.522·log₁₀(dose) - 0.8164), estimating ED₉₀ and ED₉₅ values of approximately 24 mg/kg and 41 mg/kg, respectively. These estimates informed our choice to use 30 mg/kg as a high-efficacy dose falling within the upper behavioral response window. This was further supported by internal dose-range testing (10 - 40 mg/kg), in which 30 mg/kg consistently induced robust behavioral responsiveness without signs of distress or functional impairment. The selected dose also aligns with recent reports using the structurally related compound 5-MeO-DMT, where 30 mg/kg elicited strong head-twitch responses, a validated preclinical correlate of serotonergic hallucinogen activity [53, 54].

For the group treated during the ongoing UCMS protocol (DMT-d28), the stressor was applied five hours prior to DMT administration to ensure a lower-stress baseline at the time of treatment, aligning conditions with other experimental groups. For the DMT+Iso group, mice were anesthetized with inhaled isoflurane prior to DMT injection. Anesthesia was induced at 5% and maintained at 1.5–3% (flow rate: 0.8 L/min). To ensure stable exposure and minimize variability, isoflurane was initiated 30 min before and continued for 90 min following the DMT injection.

Fluoxetine-treated mice were administered fluoxetine intraperitoneally at a dose of 20 mg/kg daily by an experienced caretaker, starting on postnatal day 42 and continuing for 30 days. Treatment concluded one day before the initiation of behavioral testing to ensure the absence of residual drug effects during testing.

Tamoxifen was administered to all groups at a dose of 100 µg/kg/day intraperitoneally for three consecutive days [55], beginning on postnatal day 48. This timing was carefully chosen to induce recombination in DCXCreERT2::TdTOM^lox/lox^ mice as the labelling window, which can occur within one day and may span several days to weeks [55, 56], will likely capture newborn neurons during the final days of UCMS exposure and the post-stress period, which are expected to integrate into hippocampal network during behavioral testing [46]. On tamoxifen injection days, all animals were housed under the same environmental conditions to reduce variability.

### UCMS protocol and confirmation of depression-like symptoms onset

The UCMS protocol is a chronic stress paradigm designed to induce depression-like behaviors through prolonged exposure to unpredictable stressors. All mice, except those in the Non-UCMS group, were subjected to the UCMS regimen. Non-UCMS mice underwent gentle handling during the same period as the stressors. The UCMS protocol was implemented with minimal modifications as described by Monteiro et al., 2015 [57], and exemplified in Supplementary Table 1. A single aversive stressor was applied daily over an 8-week period. The stressors included confinement, where mice were placed in 50 ml plastic tubes (Falcon) with openings at both ends for 90 min; agitation, involving groups of five mice placed in a plastic box on an orbital shaker set at 150 rpm for 90 min; exposure to rat feces (60 g of soiled rat bedding for 90 min, followed by cage cleaning); hot air jets from an 800 W hairdryer for 8 min, with minimal noise and a box temperature of 35–40 °C monitored by a digital thermometer; nighttime illumination (2 h of light during the dark phase); reversed light/dark cycles for two consecutive days; and inclined cage positioning at a 45° angle for 90 min. Stressors were presented in a pseudorandomized (i.e., randomized for the first group of littermates and subsequently maintained for all others to ensure consistency across experimental conditions) and unpredictable order.

Animals were randomly allocated to experimental groups prior to the start of UCMS exposure. Group assignment occurred immediately after baseline SPT (see Supplementary Fig. 1), with mice assigned to one of three initial arms: UCMS-exposed only, UCMS-exposed with DMT administration on day 28, or UCMS-exposed with fluoxetine treatment. After the UCMS period and post-UCMS SPT, UCMS-exposed-only animals were further randomized to receive a single dose of saline, DMT under isoflurane anesthesia, or DMT alone. During the initial group assignment process, care was taken to avoid placing multiple littermates or cagemates into the same treatment group whenever possible, in order to minimize potential cage-level or litter-specific confounds while preserving the integrity of randomization. In addition to balancing group assignments, we formally evaluated group-level differences in baseline sucrose preference using one-way ANOVA followed by Tukey’s post hoc test (see Supplementary Fig. 2). No significant differences in group means or variances were found, confirming that behavioral baselines were comparable prior to the onset of UCMS and treatment.

**Fig. 2:**
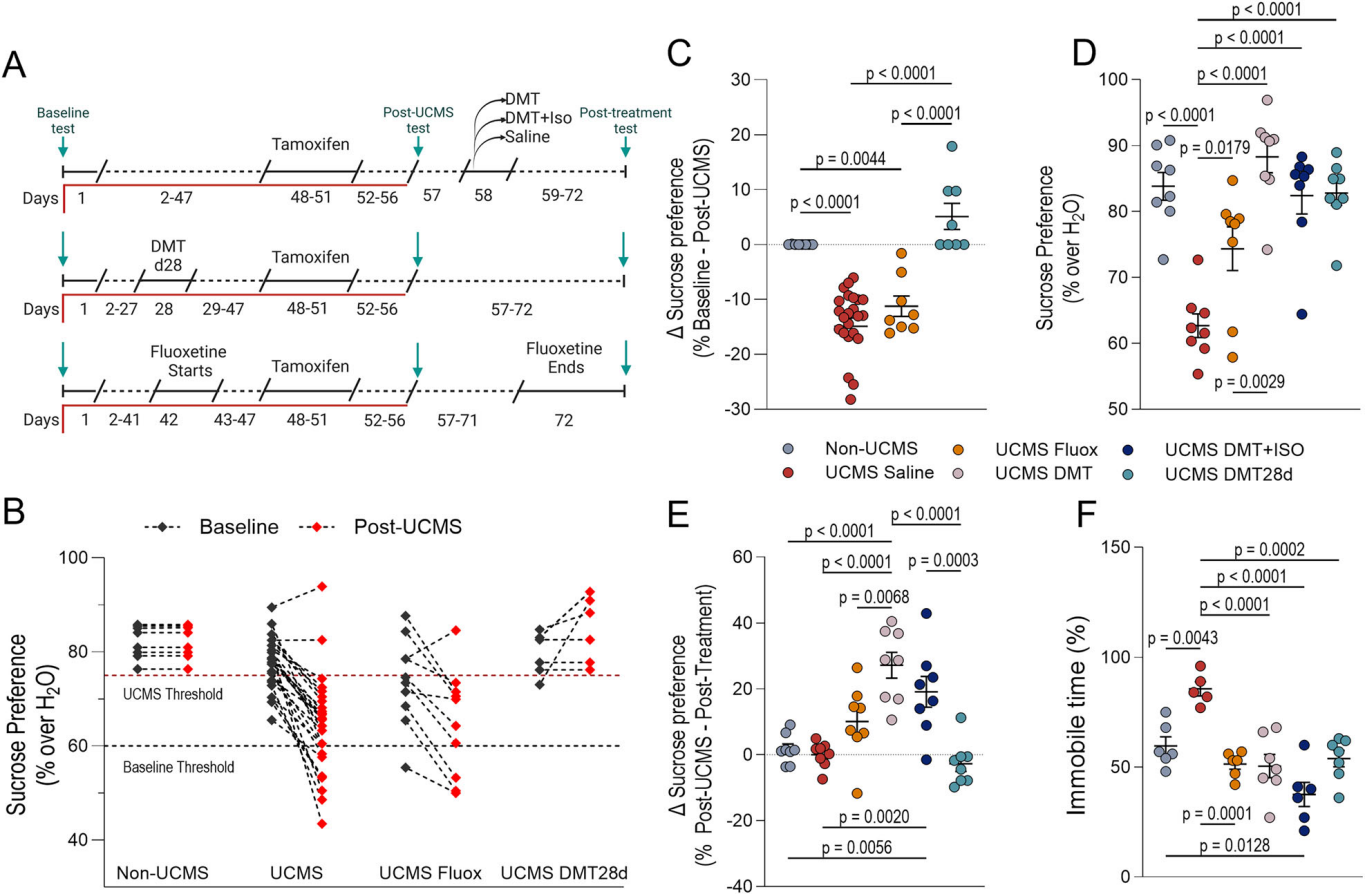
DMT and fluoxetine treatments differentially reverse UCMS-induced anhedonia and behavioral despair. **A** Experimental timeline highlighting behavioral testing and treatment periods. Green arrows indicate behavioral test timepoints. Sucrose preference tests were conducted at baseline, after UCMS exposure (pre-treatment), and after treatment across all groups. Tail suspension test was performed only at the post-treatment timepoint. Treatment regimens are the same as shown in Fig. 1A: acute DMT, DMT+Iso, or Saline on day 58; DMT-d28 during the UCMS protocol; and daily fluoxetine from days 42–71. **B** Sucrose preference before and after UCMS exposure, prior to treatment, illustrating stress-induced anhedonia across groups. A black dashed line marks the 60% baseline exclusion threshold; mice showing unusually low sucrose preference before UCMS (n = 1) were considered sucrose non-responders and excluded. A red dashed line at 75% indicates the post-UCMS exclusion threshold; mice that maintained high sucrose preference after UCMS without treatment (n = 2) were excluded from further intervention due to stress insensitivity. **C** Change in sucrose preference (Δ sucrose preference) from baseline to post-UCMS for each group shown in panel B. **D** Post-treatment sucrose preference across all groups. **E** Change in sucrose preference (Δ sucrose preference) from pre- to post-treatment. **F** Tail suspension test immobility time measured after treatment to assess behavioral despair. All data are presented as individual values with mean ± SEM (panels C–F); (**B**) shows individual values only. Analyses in (**C–F**) were conducted using one-way ANOVA followed by Tukey’s post-hoc test; adjusted p-values are shown in the figure. Sample sizes: (B, after exclusions): Non-UCMS (n = 8), UCMS (n = 21), UCMS Fluoxetine (n = 8), UCMS DMT-d28 (n = 5). (**C**): same as (**B**). (**D–E**): all groups n = 8. (**F**): Non-UCMS (n = 6), UCMS Saline (n = 5), UCMS Fluoxetine (n = 6), UCMS DMT (n = 7), UCMS DMT+Iso (n = 6), UCMS DMT-d28 (n = 7).

To confirm the development of a depressive-like phenotype, body weight and coat condition were monitored weekly, as shown in Fig. 1B-D. In addition, all animals underwent sucrose preference testing (SPT) at three time points: pre-UCMS, post-UCMS, and post-treatment (Fig. 2). Animals with extreme scores were excluded based on predefined criteria: mice with baseline sucrose preference <60% were excluded as potential non-responders to the test (n = 1); and untreated mice with post-UCMS sucrose preference >75% were excluded as stress-insensitive (UCMS-resistant) animals (n = 2). These criteria ensured that all included animals were behaviorally responsive and sensitive to the effects of UCMS.

### Behavioral experiments and analysis

Following UCMS exposure and treatment completion, behavioral tests were conducted to individually assess anhedonia, apathy, cognition, and anxiety. Behavioral scoring and assessment was performed by experienced observers (R.V.L.dC., T.S., or T.C.M.) and computational tools. All behavioral assessments were conducted by experimenters blinded to the treatment groups. The first tool was DeepLabCut (DLC), a deep-learning-based software that has shown reliable results assessing animal behavior [58]. The second tool was AnimalTA, a tracking software that allows fast processing of a high number of multi-arena videos [59]. Custom Python or MATLAB codes were developed for downstream analysis of tracking outputs and are available upon request. The selected tests were performed as follows:

The Open Field Test (OFT) consisted of placing animals in an open arena with dimensions of 40 cm (length) × 32 cm (width) × 15 cm (height) for 10 min to assess general locomotor activity and anxiety-related behavior. Animals were always placed at the center of the arena, facing the wall opposite to the behavioral room door to minimize external distractions and ensure consistency across trials. A center zone was defined as the innermost area spanning one-third of the arena’s outer dimensions in each direction, resulting in a central rectangle that represented approximately 11% of the total arena area. AnimalTA [59] was used for tracking, and the trajectories were analyzed using our custom MATLAB pipeline.

Anxiety was evaluated by the elevated plus maze test (EPM). Animals were placed in a cross-shaped maze elevated 52 cm above the ground. The maze consisted of four arms, each 62 cm (length) × 6.5 cm (width), with two opposing arms enclosed by 20 cm high walls and two open arms. Each animal was tested for 10 min. To ensure consistency and minimize external distractions, animals were always placed in the center of the maze, facing an open arm oriented opposite to the door of the behavioral room. AnimalTA was used to extract movement trajectories, which were then analyzed using the same custom MATLAB codes described above. Additionally, we manually analyzed the number of head dips outside the arena by a trained researcher.

The Sucrose Preference Test (SPT) assessed anhedonia, where mice were individually housed beginning on the habituation day and remained singly housed throughout the test period to allow accurate measurement of individual fluid intake. Animals were first habituated to drinking water from two bottles for 24 h. One of the bottles was then replaced with a 1% sucrose solution, and after 24 h, the bottle positions were switched to eliminate location bias. The total volume consumed from each bottle over the 48 h testing period was recorded by an experienced researcher, and sucrose preference was calculated as the percentage of sucrose solution consumed relative to total fluid intake.

The Tail Suspension Test (TST) evaluated behavioral despair. Animals were suspended by their tails 45 cm above the ground in a 60 cm (height) × 15 cm (depth) × 70 cm (width) square box. Their tails were gently secured with adhesive tape to a wooden shaft at a height of 45 cm. A small climb blocker, made from a 4 cm long cylinder cut from a 15 ml Falcon tube, was placed around their tails to prevent tail-climbing behavior, which is commonly observed in C57BL/6 mice. As this test requires frame-by-frame pose estimation, DeepLabCut (DLC) was used for tracking, and the outputs were analyzed with a custom-made Python script, available upon request.

**Fig. 3:**
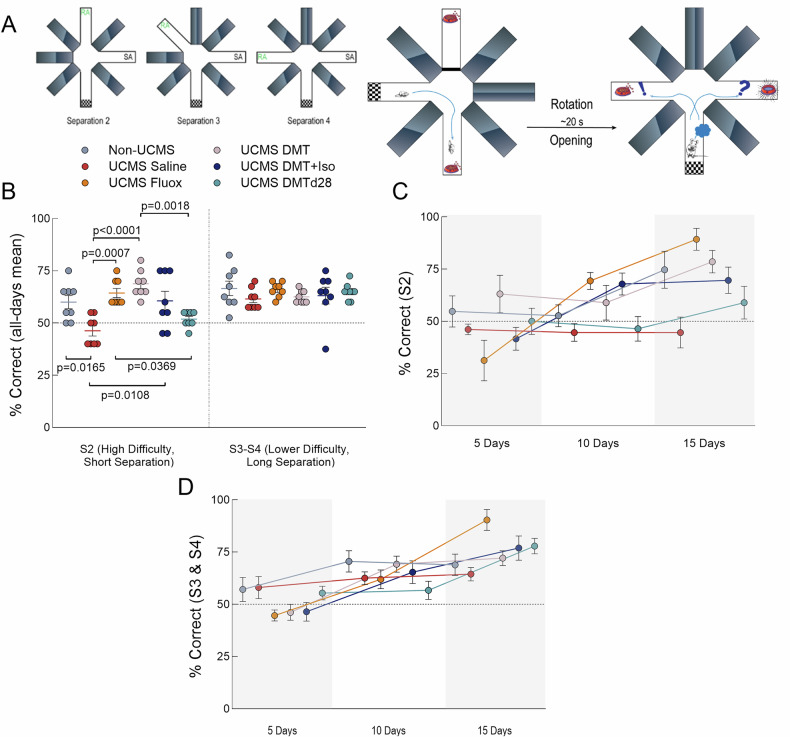
DMT reverses UCMS-induced cognitive impairments. **A** Schematic of the DNMP-RAM task. *Left*: Task difficulty is manipulated by varying the spatial separation between the sample arm (SA) and the correct choice arm, denoted as S2 (adjacent arm), S3, or S4 (maximally separated). *Right*: Each trial consists of a sample phase, where the animal is allowed to explore one open arm to retrieve a reward. After a brief delay (~20 s), the maze is rotated, and the animal is returned to the center for the choice phase, where both arms are opened and a reward is delivered only if the animal enters the non-matching arm. **B** Task performance across groups. *Left panel*: Significant group differences were found for the most difficult condition (S2). UCMS-saline mice showed impaired performance relative to Non-UCMS controls, while animals treated post-UCMS with fluoxetine, DMT, or DMT+Iso performed significantly better than UCMS-saline. *Right panel*: No significant group differences were detected under lower difficulty (S3 and S4) conditions. **C** Learning trajectories for high-difficulty (S2) trials across the 15-day testing period. Analysis showed significant effects of treatment and training. Non-UCMS, Fluoxetine-, DMT-, and DMT+Iso-treated animals exhibited significantly better performance at the final timepoint compared to UCMS-saline **D** Learning trajectories for lower difficulty (S3/S4) trials. A significant effect of training was observed (p < 0.0001), but no group differences were detected. Analyses in panels were conducted using either one-way ANOVA followed by Tukey’s post-hoc test (in B), or repeated-measures two-way ANOVA with Holm-Sidak post-hoc test (in C and D). All data are presented as individual values with mean ± SEM. Sample size: all groups, n = 8.

The Radial Arm Maze Test (RAM) assessed cognitive function, specifically reference and working memory. The maze consisted of eight arms, each 30 cm (length) × 8 cm (width) × 30 cm (height), connected to a central round platform with a 20 cm diameter. Each arm was equipped with removable doors and small wells at the end for food rewards, which were kept out of sight. The Delayed Non-Matching to Place (DNMP) paradigm was employed, consisting of sample and test phases. During the sample phase, the mouse was confined in the start arm without a reward while a single reward (a loop of Fruit Loops™) was placed in a designated sample arm located two arms away. Other arms remained closed, and the animal was allowed to retrieve the reward within four minutes. During the test phase, the mouse was returned to the start arm, and a reward was placed in a new arm. The mouse had to distinguish the previously visited sample arm from the newly opened reward arm to locate the food. A correct first attempt was recorded as a success, while errors were allowed to be corrected within 10 min, though errors were still noted. To minimize reliance on olfactory cues, the maze was rotated between phases (~20 s), and the apparatus was thoroughly cleaned between subjects. The location of the reward arm was rotated relative to the sample arm by a factor of S# (where S represents the separation and # denotes the number of arms separating the two open arms). The test was repeated 20 times per condition (4 trials/day over 15 days) for each separation condition (S2, S3, and S4), resulting in 60 trials per condition per subject, as exemplified in Fig. 3A. Animals with deficits in pattern separation were expected to show difficulty specifically in the higher difficulty S2 condition. To ensure task motivation, animals had limited access to food during the 15 days of testing and were habituated to the reward five days prior to maze training. All data were recorded manually during the experiment by an experienced researcher blinded to the treatment groups.

The order and timing of behavioral assessments were carefully designed to balance sensitivity, feasibility, and interpretability across affective and cognitive domains (Supplementary Fig. 1). SPT was performed at three key stages: before stress (baseline SPT), after UCMS but prior to UCMS-only group treatment (post-UCMS SPT), and after the UCMS-only group treatment period (post-treatment SPT). This allowed us to monitor the onset and potential reversal of anhedonia over time. Following treatment allocation, animals underwent the DNMP-RAM protocol continuously for 15 days to assess cognitive function. To maximize data yield while minimizing the impact of behavioral interference, affective tests were conducted in parallel with the RAM protocol at specific timepoints. The OFT was performed on day 4 to verify that treatment effects on cognition or despair could not be attributed to changes in general locomotion or anxiety-related behavior. The TST was conducted on day 7, under the assumption that a short exposure to this relatively brief task would not exert long-lasting effects on learning performance. The post-treatment SPT, conducted between days 10 and 13, was intentionally delayed to capture sustained recovery of hedonic behavior rather than transient post-treatment effects. Given its passive and home-cage-based nature, SPT was not expected to interfere with RAM learning or motivation. Finally, the EPM was conducted on day 15, immediately after the last RAM session, to avoid any influence of maze novelty or stress on anxiety-like behavior. This schedule enabled robust, temporally distributed assessment across behavioral domains while preserving the integrity of each test’s interpretability.

### Tissue processing and histological analysis

Following the completion of behavioral experiments, animals were euthanized on day 78 via an overdose of Ketamine Hydrochloride (150 mg/kg) combined with Xylazine (8 mg/kg). They were then positioned in dorsal decubitus and perfused transcardially with 30 mL of phosphate-buffered saline (PBS), followed by 30 mL of 4% paraformaldehyde (PFA). After perfusion, craniotomies were performed to extract the brains, which were then submerged in 4% PFA at 2–8 °C overnight for fixation. The following day, brains were cryoprotected by immersion in 30% sucrose solution for 24 h. Once cryoprotected, they were briefly rinsed in 0.1 M phosphate buffer (PB), rapidly frozen by immersion in −80 °C isopentane, and then stored at −80 °C before further processing.

Frozen brains were horizontally sectioned into 25 µm-thick slices using a cryostat. Sections were collected on gelatin-coated slides in an alternating series, with a 200 µm spacing between slices, covering the entire hippocampal region (AP 2.6 −4.4 mm, according to Paxinos, 2012). Tissue sections were mounted with Fluoromount-G™ medium containing DAPI (ThermoFisher, cat. 00-4959-52) and examined under a ZEISS reflected light microscope. Stereoinvestigator software (MBF Bioscience) was used to assist in cell quantification. tdTOM+ cells were manually counted in both hippocampi by an experimenter blinded to the experimental groups.

Ectopic cells were defined as tdTOM+ cells located more than 20 μm away from the granule cell layer (GCL) in any direction. This threshold was selected based on prior literature [60] and chosen to ensure a conservative and unambiguous criterion for identifying aberrantly migrating cells. In practice, this distance corresponds to approximately two cell body widths of intervening space, which consistently excluded neurons integrated within the GCL. Although some studies have used lower cutoffs, such as 10 μm [61], the 20 μm threshold provided greater confidence that only clearly displaced cells were classified as ectopic.

Importantly, although ketamine has been associated with neuroplastic effects, its use at the time of euthanasia is unlikely to have influenced our histological outcomes. The tamoxifen labeling period had concluded well before perfusion, and any ketamine-induced changes would have occurred outside the relevant integration window. Moreover, since all animals were euthanized under identical conditions, any potential effects would not have introduced group-specific bias.

### Statistical analysis

All data were tested for normality using D’Agostino and Pearson’s omnibus normality test and were confirmed to follow a normal distribution. The sample size varies across some tests due to technical errors during behavioral recordings or equipment failures, which resulted in the loss of specific trials while the affected animals remained in the study for all other assessments. Data are expressed as Mean ± SEM, unless stated otherwise. Statistical significance was set at p < 0.05. Group comparisons were conducted using one-way ANOVA followed by Tukey’s multiple comparisons test, or two-way ANOVA followed by Holm-Sidak’s multiple comparisons test. All statistical analyses were performed using GraphPad Prism 10 (San Diego, CA, USA) and csv processing software.

### Ethical considerations

All procedures were conducted in accordance with the ethical guidelines set by the Brazilian National Council for the Control of Animal Experimentation (CONCEA). The protocols were approved by the institutional animal care and use committee of the Federal University of Rio Grande do Norte (approval numbers 036/2019 and 186.036/2019).

## Results

### Depression-like phenotypes

Mice were exposed to 56 days of UCMS before receiving a single injection of either DMT, to test if acute psychedelic treatment is able to reverse UCMS-induced phenotypes; DMT under isoflurane anesthesia (DMT+Iso), to examine the effects of conscious psychedelic experience; or vehicle control (saline). In parallel, we administered DMT during the stress period (DMT day 28) to assess whether treatment administered in the midst of UCMS exposure could still exert behavioral and neurogenic effects. Finally, a positive control group was treated with daily fluoxetine, a conventional antidepressant, for 30 days, partially overlapping with the UCMS protocol (Fig. 1A).

Consistent with previous findings, weekly monitoring revealed that UCMS-exposed mice had significantly deteriorated coat state and reduced weight gain compared to Non-UCMS controls (Fig. 1B-D), both established indicators of stress-induced behavioral alterations. Specifically, two-way ANOVA of coat scores indicated significant main effects of time, treatment, and their interaction (all p < 0.0001). Post-hoc analysis (Holm-Sidak multiple comparison test) at the final measurement confirmed that all UCMS groups showed significantly worse coat conditions than Non-UCMS controls (p < 0.0001 for UCMS and UCMS Fluoxetine; p = 0.0004 for UCMS DMT-d28). Interestingly, mice receiving DMT midway through the stress protocol (UCMS DMT-d28) showed a modest yet significant improvement in coat state compared to both UCMS alone (p = 0.0042) and UCMS Fluoxetine groups (p = 0.0012). Notably, fluoxetine-treated UCMS mice displayed the worst coat condition among stressed groups, significantly worse even compared to UCMS alone (p = 0.0020). Moreover, two-way ANOVA analysis of weight gain showed significant effects of time and the interaction between time and treatment (both p < 0.0001), but no main effect of treatment alone. To clarify these differences, a subsequent one-way ANOVA was conducted on the total weight change (Δ weight) during the UCMS protocol (Fig. 1F), revealing a significant difference among groups (p < 0.0001). Post-hoc analysis (Tukey’s multiple comparisons test) demonstrated that all UCMS groups experienced significantly reduced weight gain compared to Non-UCMS controls (Non-UCMS vs. UCMS, p < 0.0001; Non-UCMS vs. UCMS Fluoxetine, p < 0.0001; Non-UCMS vs. UCMS DMT-d28, p = 0.0002). However, no significant differences were found among the UCMS groups themselves (UCMS vs. UCMS Fluoxetine, p = 0.8992; UCMS vs. UCMS DMT-d28, p = 0.9083; UCMS Fluoxetine vs. UCMS DMT-d28, p = 0.6990), indicating that this protocol had consistent effects across groups.

To evaluate reward-seeking behavior, sucrose preference tests (SPTs) were conducted before and after UCMS exposure, as well as after treatments (Fig. 2A). To first assess the impact of UCMS itself, SPT results were compared across the stress-exposed cohorts prior to any treatment (Fig. 2B). Then, we performed one-way ANOVA analyses followed by Tukey’s multiple comparisons test of sucrose preference change between the two assessments (Fig. 2C). UCMS-exposed mice exhibited a reduced sucrose preference relative to Non-UCMS controls (p < 0.0001), confirming the anhedonic phenotype in the cohort used for downstream analyses. Notably, mice in the UCMS DMT-d28 group, treated during the stress period, showed significantly less anhedonia in relation to the untreated UCMS group (p < 0.0001), and were comparable to Non-UCMS controls (p = 0.3723). In contrast, initiating fluoxetine treatment during the later stages of UCMS did not prevent the development of anhedonia, as sucrose preference remained significantly lower than Non-UCMS levels (p = 0.0044) and similar to that of the untreated UCMS group (p = 0.4933).

We then assessed changes in sucrose preference between the end of the UCMS protocol and after treatment administration (Fig. 2D). All DMT-treated groups, including DMT alone, DMT under isoflurane, and DMT-d28, exhibited significantly higher sucrose preference compared to UCMS saline controls (all p < 0.0001), consistent with a partial or full reversal of anhedonia. Fluoxetine produced a modest but significant increase (p = 0.0179), though sucrose preference remained lower than in DMT-treated animals (p = 0.0029). Further analysis of pre- to post-treatment changes (Fig. 2E) confirmed no significant differences in Non-UCMS or UCMS saline groups, whereas both DMT (p < 0.0001) and DMT+Iso (p = 0.0020) groups showed marked improvements. Interestingly, the DMT-d28 group displayed no significant change at this time point (p = 0.9905 vs. UCMS saline), suggesting that DMT administration at day 28 may have already exerted its maximal effect in reversing UCMS-induced anhedonia by the time of the post-UCMS assessment.

To evaluate behavioral despair phenotype, we employed the TST (Fig. 2F). Since this test requires frame-by-frame pose estimation, we used DeepLabCut for tracking. A custom Python script was developed to analyze the DLC output controls (p = 0.0043), reflecting the development of a depressive-like state. Remarkably, all tested treatments, i.e., DMT (p < 0.0001), DMT+Iso (p < 0.0001), fluoxetine (p = 0.0001), and DMT-d28 (p = 0.0002), effectively reduced immobility compared to UCMS saline controls. Despite targeting different timepoints and mechanisms, these interventions produced similarly robust effects in reversing helplessness behavior. No significant differences were detected among the treatment groups themselves (all p > 0.09), suggesting convergent efficacy in this assay. Taken together with the sucrose preference data, these results support a broad antidepressant-like action of DMT and highlight its potential to counteract distinct dimensions of depression-related behavior.

### Anxiety-like phenotypes

In the OFT (Supplementary Fig. 3A–C), we did not observe any statistically significant differences between UCMS saline-treated mice, Non-UCMS controls, or any of the treatment groups following one-way ANOVA analysis of the distance travelled. Similarly, no significant differences were found among groups when measuring time spent in the center, expressed as a percentage of total test duration. Representative tracking paths and occupancy heatmaps from a sample animal are shown in Supplementary Fig. 3A and illustrate the spatial distribution of exploration across the arena.

Likewise, in the EPM (Supplementary Fig. 3D–F), ANOVA analysis revealed no significant differences across treatment groups, UCMS saline, or Non-UCMS controls in terms of the percentage of time spent in the open arms. We also found no significant differences in the number of head dips, an index of exploratory behavior. A representative example of the movement trajectory in the EPM is shown in Supplementary Fig. 3D, highlighting typical arm entries and tracking across arms. Taken together, these results suggest that neither UCMS nor any of the pharmacological treatments tested induced measurable changes in anxiety-like behavior under the current experimental conditions.

### Cognitive phenotypes

To assess cognitive function and specifically examine pattern separation, we employed the DNMP-RAM task, illustrated in Fig. 3A. Performance analysis for lower difficulty, longer separation conditions (S3 and S4) revealed no significant differences across groups (Fig. 3B, right panel). However, significant group differences emerged at the higher difficulty, lower separation level (S2) of the task (Fig. 3B, left panel). Specifically, ANOVA followed by Tukey’s multiple comparisons test showed that UCMS-saline mice performed significantly worse than naïve controls (Non-UCMS, p = 0.0165). Moreover, animals treated with fluoxetine (p = 0.0007), DMT (p < 0.0001), and DMT+Iso (p = 0.0108) displayed significantly enhanced performance compared to the UCMS-saline group, indicating a reversal of UCMS-induced cognitive deficits. Conversely, the DMTd28 group did not significantly differ from saline-treated animals (p = 0.7273), indicating no cognitive improvement. We further analysed the learning trajectory across the 15-day period to determine if groups had distinct learning curves during the higher difficulty separation trials (S2). As illustrated in Fig. 3C for the higher difficulty task, animals from the UCMS-saline and DMTd28 groups showed minimal to no learning throughout the assessment period, plateauing around the chance-level correct-response rate. Repeated-measures two-way ANOVA analysis showed significant differences between treatments (p < 0.0001) and significant effects of training (p < 0.0001). Mice treated with fluoxetine, DMT, and DMT combined with isoflurane post-UCMS, as well as the Non-UCMS group, exhibited significantly higher learning than UCMS-Saline at the 15-day mark (Holm-Sidak multiple-comparisons test; p = 0.0001, p = 0.0016, p = 0.0169, and p = 0.0049, respectively). Similar analysis of lower difficulty separation trials S3 and S4 (Fig. 3D) revealed a significant effect of training (p < 0.0001), but not of treatment (p = 0.7677).

### Adult neurogenesis and aberrant GC integration

**Fig. 4:**
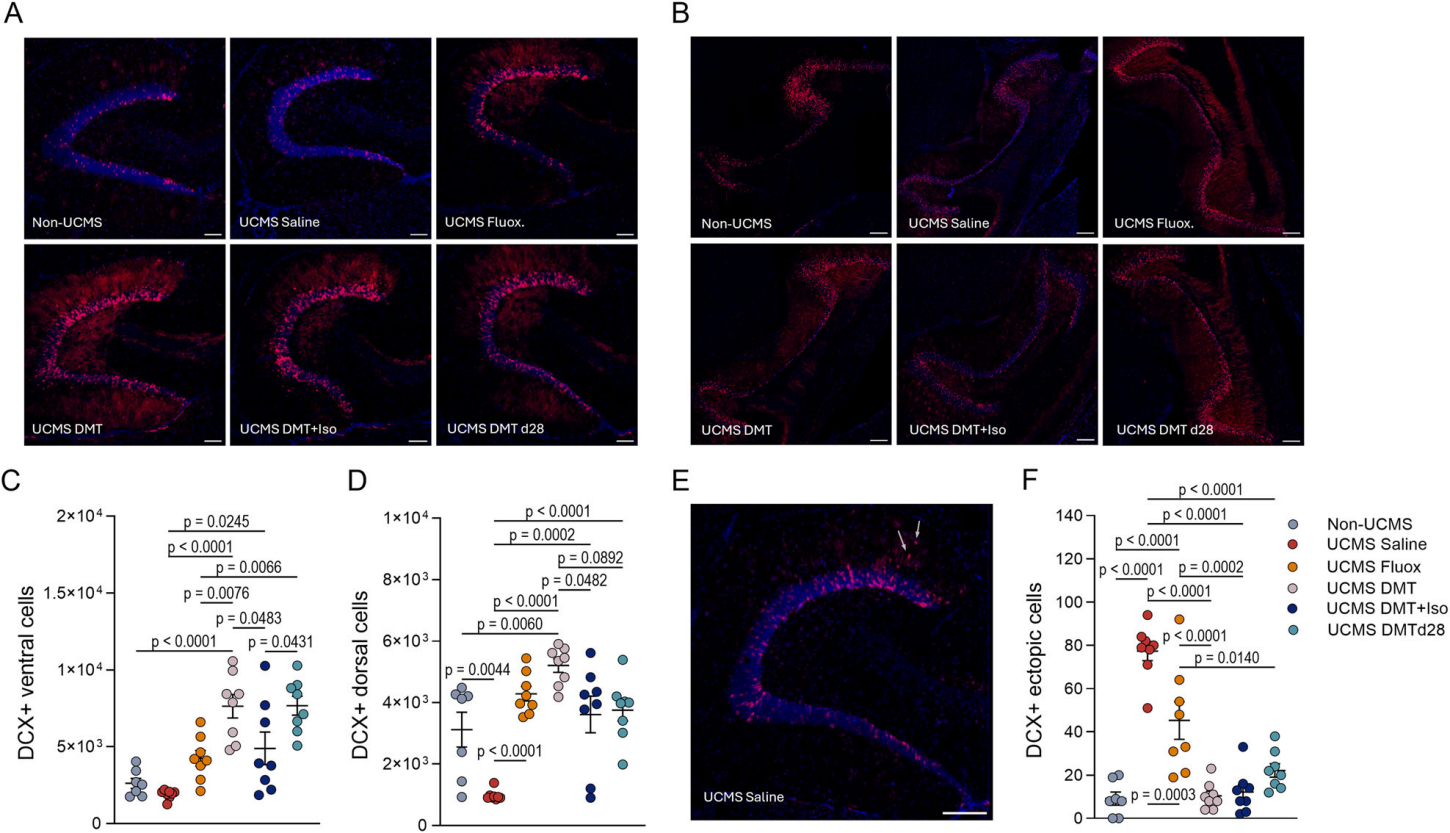
DMT promotes adult hippocampal neurogenesis and restores proper granule cell integration. **A, B** Representative confocal images of DCX-TOM⁺ granule cells in the dorsal (**A**) and ventral (**B**) DG across experimental groups. Scale bars: 80 μm (A), 200 μm (B). DCX⁺ newborn granule cells (magenta) were labeled by tamoxifen-induced recombination in DCXCreERT2::TdTOM^lox/lox^ mice. **C** Quantification of ventral DCX-TOM⁺ cells revealed significantly increased neurogenesis in DMT- and DMT-d28–treated mice compared to Non-UCMS controls, with both groups also surpassing DMT+Iso and fluoxetine. **D** In the dorsal DG, UCMS-saline animals showed reduced neurogenesis compared to Non-UCMS controls. All treatments led to significantly increased neurogenesis compared to UCMS-saline, with DMT-treated animals showing the strongest response. **E** Representative image of ectopic DCX-TOM⁺ cells in a UCMS-saline animal. White arrows indicate DCX⁺ cells located outside the granule cell layer. Scale bar: 100 μm. **F** Quantification of ectopic DCX-TOM⁺ cells. UCMS-saline animals exhibited a marked increase in ectopic cells compared to Non-UCMS, indicating disrupted neuronal integration. All treatments significantly reduced ectopic neurogenesis relative to UCMS-saline. All data are presented as individual values with mean ± SEM. Analyses were performed using one-way ANOVA followed by Tukey’s post-hoc test. Sample sizes: Non-UCMS (n = 7), all other groups (n = 8).

To evaluate the impact of treatments on GCs’ neurogenesis and their integration within the DG, we performed confocal microscopy of newborn GCs by the tamoxifen-induced tagging within the genetically modified DCXCreERT2::TdTOM^lox/lox^ mice (Fig. 4A, B). Briefly, following behavioral assessments, animals were euthanized, and their brains were collected for detailed histological analysis. DCX-TOM+ cells were systematically quantified along the dorsoventral hippocampal axis, with additional consideration given to ectopically located neurons (i.e., cells integrating outside the granule cell layer, GCL). Histological assessment with one-way ANOVA analyses revealed significant treatment-related differences in the number and distribution of DCX-TOM+ neurons. In the ventral hippocampus (Fig. 4C), both the DMT and DMTd28 groups exhibited roughly a two-fold increase in DCX-TOM+ cell counts compared to non-stressed control animals (Non-UCMS vs. DMT p < 0.0001; vs DMTd28 p < 0.0001). These two DMT-treated groups did not differ from one another (p > 0.9999), but each showed significantly greater neurogenesis than fluoxetine-treated mice (p = 0.0076 and p = 0.0066 for DMT and DMT-d28 vs. Fluoxetine, respectively), as well as those receiving DMT under isoflurane (p = 0.0483 and p = 0.0431). DMT+Iso was still significantly more effective than UCMS-saline (p = 0.0245), while fluoxetine treatment did not have a significant effect (p = 0.1288).

Additionally, analyses within the dorsal hippocampus (Fig. 4D) revealed that UCMS-saline animals showed reduced neurogenesis compared to Non-UCMS controls (p = 0.0044), indicating that chronic stress suppressed neurogenic activity in this region. Moreover, all treatments led to markedly increased neurogenesis relative to UCMS-saline animals, including DMT (p < 0.0001), DMT+Iso (p = 0.0002), fluoxetine (p < 0.0001), and DMT-d28 (p < 0.0001). DMT-treated mice exhibited the strongest neurogenic response and were the only group to significantly exceed Non-UCMS levels (p = 0.0060), suggesting not only a reversal of stress-induced deficits but a net increase in neurogenesis. DMT-treated animals also differed significantly from those given DMT under isoflurane (p = 0.0482), pointing to a possible role of wake psychedelic experience in neurogenesis. Fluoxetine and DMT-d28 groups showed intermediate DCX-TOM⁺ cell counts, although neither differed significantly from the DMT group (p = 0.5197 and p = 0.0892, respectively), suggesting a moderate, yet measurable, effect of these treatments on dorsal neurogenesis.

We also examined the incidence of ectopic DCX-TOM⁺ cells, indicative of disrupted neuronal integration due to stress (Fig. 4E, F). Saline-treated UCMS mice displayed a significant increase in ectopic cell numbers compared to Non-UCMS controls (p < 0.0001), reflecting aberrant neurogenesis. Strikingly, all treatment groups showed significantly reduced ectopic cell counts relative to UCMS-saline, including DMT (p < 0.0001), DMT+Iso (p < 0.0001), fluoxetine (p = 0.0003), and DMT-d28 (p < 0.0001). Among these, DMT and DMT+Iso reduced ectopic cell incidence to levels statistically indistinguishable from Non-UCMS animals (p > 0.9999 and p = 0.9979, respectively), suggesting near-complete normalization. In contrast, fluoxetine- and DMT-d28-treated mice did not fully normalize ectopic integration and still differed significantly from Non-UCMS (p < 0.0001 and p = 0.4223, respectively). Furthermore, both DMT and DMT+Iso outperformed fluoxetine in reducing ectopic neurogenesis (p < 0.0001 and p = 0.0002), and fluoxetine in turn was significantly more effective than DMT-d28 (p = 0.0140). No significant differences were observed between the DMT and DMT+Iso groups (p = 0.9998), nor between either of those and DMT-d28 (p = 0.4949 and p = 0.6616). These findings highlight a superior efficacy of DMT-based interventions, particularly when administered after UCMS, in restoring proper neuronal integration patterns.

### Ectopic GC counts predict behavioral deficits

**Fig. 5:**
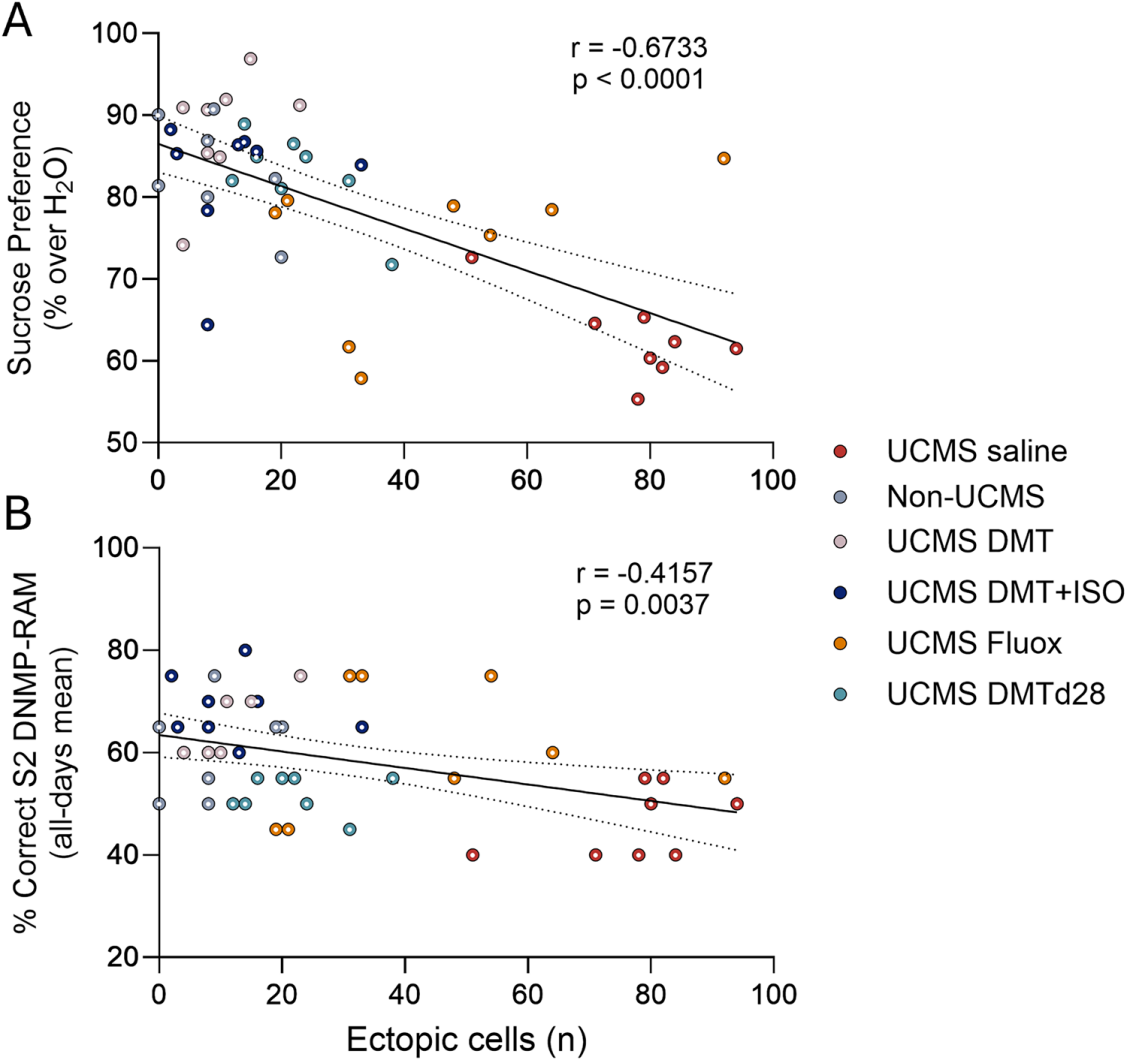
Ectopic granule cell counts negatively correlate with behavioral performance. **A, B** Correlation analyses between the number of ectopic DCX-TOM⁺ granule cells and individual behavioral outcomes across groups. **A** Post-treatment sucrose preference scores were inversely correlated with ectopic cell counts (Pearson r = –0.6733, R² = 0.4534, ****P < 0.0001). **B** Similarly, performance in the delayed non-match to place radial arm maze (DNMP-RAM) task under the most difficult condition (S2) was negatively correlated with ectopic cell number (Pearson r = –0.4157, R² = 0.1728, **P = 0.0037). All data are presented as individual values. Analyses were conducted using Pearson correlation across all subjects (n = 47).

Given the strong effects of UCMS and subsequent treatments on ectopic neurogenesis, we next investigated whether variation in ectopic DCX-TOM⁺ granule cell numbers could account for individual differences in behavioral outcomes across animals (Fig. 5). To this end, we performed Pearson correlation analyses using matched histological and behavioral data across all intervention groups. A significant negative relationship was observed between post-treatment sucrose preference and the number of ectopic cells (Fig. 5A, r = –0.6733, R² = 0.4534, p < 0.0001), indicating that animals with increased aberrant integration of abGCs exhibited more severe anhedonic behavior. A similar pattern was found in the spatial working memory task, where ectopic cell number was negatively correlated with performance in the high-difficulty (S2) DNMP-RAM condition (Fig. 5B, r = –0.4157, R² = 0.1728, p = 0.0037), suggesting impaired hippocampal-dependent cognition in animals with higher levels of disrupted neurogenesis. Together, these findings suggest that ectopic integration of abGCs may contribute to the pathophysiology underlying both affective and cognitive disturbances in the UCMS model.

## Discussion

This study presents, to our knowledge, the first preclinical investigation into the antidepressant potential of a single dose of DMT using the UCMS paradigm in mice, a validated rodent model that mimics core features of MDD, including anhedonia, behavioral despair, and cognitive impairments [48, 62]. In doing so, we address several open questions concerning DMT’s efficacy in reversing stress-induced depressive-like phenotypes, its effects on adult hippocampal neurogenesis, and the necessity of the conscious psychedelic experience for its therapeutic impact.

Although long-term stress paradigms pose logistical challenges, the UCMS model provides a more nuanced and translationally relevant framework for investigating depression than the predominantly anxiety-based assays commonly employed in psychedelic research [43, 63]. Indeed, most preclinical studies with psychedelics have prioritized anxiety-like behaviors, relying on rapid, reflexive tests that are simple to implement, require minimal training, and offer high-throughput readouts[48, 64]. While valuable, these models fall short of capturing the multifaceted nature of depression, which involves sustained behavioral, cognitive, and neurogenic disruptions [65, 66]. The present work bridges this methodological gap by employing an ethologically grounded model of depression to assess the therapeutic impact of DMT across behavioral and neurogenic domains.

To ensure the experimental treatments targeted a depressive-like phenotype, animals that retained high sucrose preference (>75%) following UCMS, indicative of stress resilience, were excluded from post-stress treatment allocation. This strategy enhanced the precision of our treatment effect estimates by focusing on animals with confirmed anhedonia, although it limits the ability to generalize our findings to stress-resilient individuals. We tested three DMT treatment regimens: a single post-stress dose, an early-dose administered midway through stress, and a dose delivered under isoflurane anesthesia. Chronic fluoxetine served as a positive control.

Our model successfully induced a robust depressive-like state, as evidenced by classic phenotypic hallmarks, such as reduced sucrose preference, worsened coat condition, increased immobility in the TST, and impaired pattern separation in the DNMP test. Moreover, UCMS-induced alterations were accompanied by reduced abGC production and abnormal ectopic integration of newborn neurons, consistent with previous findings linking stress to hippocampal disorganization and neurogenic disruption [4, 48, 62]. Interestingly, fluoxetine-treated animals displayed the poorest coat condition among stressed groups, which may reflect either drug-specific effects or the cumulative burden of daily handling required for chronic administration. While no significant anxiety-like behaviors were observed in the open-field or elevated plus-maze, this outcome aligns with prior studies showing that C57BL/6 mice, which share the genetic background of our transgenic line, exhibit limited anxiogenic responses to UCMS in these assays [51]. Nevertheless, these results indicate that none of our treatments exacerbate anxiety-related behaviors and serve as behavioral controls for a broader interpretation of our findings.

Notably, administration of DMT after UCMS reversed all core depressive-like phenotypes. Treated mice exhibited restored sucrose preference, reduced immobility in the TST, and recovered pattern separation ability. These behavioral improvements were accompanied by significantly increased abGC production in both the dorsal and ventral DG, exceeding non-stressed levels in the dorsal DG. Furthermore, DMT normalized the rate of ectopic abGC integration, contrasting with fluoxetine’s partial effects. These findings echo and extend previous reports that serotonergic psychedelics can induce rapid and sustained antidepressant-like effects in rodent chronic stress models, including UCMS [67]. Our data align with these studies not only behaviorally, but also at the level of hippocampal circuit repair, adding to the growing evidence that neurogenesis is a key mediator of psychedelic-induced resilience.

Additionally, DMT administration during the UCMS protocol (DMT d28) was associated with partial protection against the development of depressive-like behaviors. These animals exhibited preserved sucrose preference and reduced immobility in the TST, although cognitive performance in the DNMP task remained impaired. Histological analysis revealed enhanced neurogenesis relative to untreated UCMS controls and a reduction in ectopic cell integration, albeit to a slightly lesser extent than that observed in the post-stress DMT group. Given that tamoxifen was administered to all animals near the end of the UCMS protocol, approximately twenty days after DMT d28 exposure, the resulting labeling is most likely to reflect neuronal populations generated during the early post-stress recovery period rather than during the stress exposure itself. Thus, while a partial preventive role of DMT cannot be excluded, the observed effects in the DMT-d28 group are more parsimoniously interpreted as reflecting sustained neurogenic and behavioral impact following DMT administration, rather than direct protection of hippocampal neurogenesis during the stress period itself. This interpretation is supported by the relatively weaker effects observed in this group compared to post-stress DMT treatment and aligns with prior work showing that psychedelics can induce persistent plasticity-related changes [68]. Altogether, these findings suggest that a single psychedelic intervention during ongoing stress may confer long-lasting neurogenic and behavioral benefits, though such effects may be insufficient to fully counteract UCMS-induced hippocampal dysfunction.

A key question in psychedelic research concerns the necessity of subjective psychedelic experiences for therapeutic outcomes [49]. Here, we aimed to provide preliminary evidence that DMT’s effects are compatible with an anesthetized state by using isoflurane to suppress conscious experience. Notably, animals in the DMT+Iso group exhibited antidepressant-like behavioral outcomes and increased neurogenesis, which were comparable to those observed in the fully awake DMT group. These findings indicate that DMT’s effects can occur even under anesthesia. Nonetheless, these exploratory findings should be interpreted with caution. Isoflurane has been shown to engage TrkB signaling and exert antidepressant-like effects in rodents [69], but it has also been reported to suppress hippocampal progenitor proliferation and impair cognitive performance, particularly in young rodents [70, 71]. In our study, the absence of an isoflurane-only group prevents definitive attribution of the observed effects to DMT alone. However, the direction of change, with enhanced neurogenesis and improved cognitive outcomes, contrasts with the typical profile of isoflurane itself. Therefore, while our results suggest that DMT’s effects persist under anesthesia, potential interaction effects between DMT and isoflurane cannot be ruled out.

Future studies using anesthetics without known antidepressant properties, such as halothane [72], and including anesthetic-only control groups, will be critical to isolate the role of consciousness in psychedelic drug action. Additionally, combining this approach with molecular and circuit-level assays may further clarify whether distinct signaling pathways are engaged under conscious versus unconscious states. Although preliminary, our findings contribute to a growing body of evidence indicating that psychedelics may exert therapeutic effects via neuroplastic mechanisms that do not strictly require subjective experience. For instance, recent studies show that psilocybin and LSD retain therapeutic efficacy in rodents even when 5-HT2A receptor signaling is pharmacologically blocked, provided neurotrophic signaling remains intact [67, 68]. These observations have important implications for the development of next-generation, non-hallucinogenic psychoplastogens.

Taken together, our data support a growing recognition of the functional relevance of adult-born neurons in modulating the antidepressant effects of DMT [20]. Although abGCs do not project beyond the hippocampus, they are thought to influence broader stress-related networks by reshaping how the dentate gyrus encodes environmental cues [73]. The hippocampus serves as a computational hub for detecting and contextualizing stress-related stimuli, and adult neurogenesis biases this system toward enhanced pattern separation, cognitive flexibility, and inhibition of inappropriate fear generalization. Notably, suppression of neurogenesis has been shown to amplify HPA axis reactivity to stress [74], suggesting that hippocampal neurogenesis contributes to negative feedback regulation of stress physiology. Within this framework, DMT-induced increases in DG neurogenesis may help normalize both emotional behavior and physiological stress responses. Moreover, a key finding in our study was the presence of ectopic granule cells in the DG of UCMS-exposed mice, which were significantly reduced by all treatment conditions. Ectopic migration of newborn neurons has been described in models of epilepsy and chronic stress, and is thought to disrupt the sparse firing patterns essential for pattern separation in the DG [61, 75]. Computational models have shown that even a small population of these cells can impair DG-CA3 computations by creating aberrant excitatory feedback loops [61].

In our study, correlation analyses revealed that higher numbers of ectopic abGCs significantly predicted reduced sucrose preference and poorer DNMP task performance, supporting the hypothesis that improper integration of newborn neurons contributes directly to affective and cognitive deficits. Importantly, while the correlation with sucrose preference was strong, that with cognitive performance was less pronounced. Despite the modest effect size, such correlations may still yield mechanistic insight, particularly in the context of complex behavioral traits shaped by multifactorial neurobiological and environmental influences. Whether ectopic neurogenesis plays a mechanistic role in cognitive and affective dysfunction, or reflects a correlated downstream consequence, remains to be elucidated.

Chronic fluoxetine administration produced comparable cognitive rescue, but its behavioral and neurogenic profiles diverged from those of DMT. Fluoxetine yielded only partial recovery of the sucrose preference phenotype, did not significantly enhance neurogenesis in the ventral hippocampus, and failed to fully revert the aberrant ectopic integration of abGCs. In contrast, DMT markedly increased neurogenesis across both hippocampal poles and nearly normalized aberrant integration. While our study did not directly examine molecular pathways, prior research implicates downstream signaling as central to synaptic remodeling, neuronal maturation, and circuit integration, and it is widely associated with the efficacy of both classical and fast-acting antidepressants. Thus, our findings suggest that DMT’s effects may involve not only 5-HT2A receptors, but also 5-HT2B and 5-HT2C receptors, both of which modulate plasticity and neurogenesis [22], or other neurotrophic signaling pathways [68]. Future studies should incorporate molecular analyses to clarify the contribution of these pathways. Measuring markers such as BDNF, phospho-TrkB, mTOR, p70S6K, and CREB in specific hippocampal subregions could elucidate the intracellular cascades mediating DMT’s neurogenic and behavioral effects. Complementary approaches, such as the use of pharmacological antagonists or conditional genetic disruptions of key receptors or signaling nodes, will be critical for isolating causal mechanisms and guiding the development of safer, non-hallucinogenic analogs.

Neuroinflammatory processes may further shape the permissiveness of the neurogenic niche. Chronic stress elevates proinflammatory cytokines such as TNF-α and IL-1β, which impair neurogenesis and dendritic complexity [76], while anti-inflammatory cytokines like IL-10 have been shown to rescue cognition and spine density in depression models [77]. Microglia are central mediators of these effects through the release of cytokines, phagocytosis, and interactions with neural progenitors [78]. Although it remains unknown whether DMT directly modulates microglial activity or cytokine signaling, studies on *ayahuasca* suggest that its constituents, including DMT, can attenuate neuroinflammation and associated anxiety-like behaviors [79, 80]. These anti-inflammatory actions may contribute to the preservation of neurogenesis observed in our stress-exposed mice. Ultimately, the behavioral efficacy of enhanced neurogenesis likely depends not only on the production of abGCs but also on a supportive microenvironment during their maturation window [81].

Moreover, in this study, we restricted our experiments to male mice, as the UCMS paradigm does not consistently elicit robust depressive-like phenotypes in C57BL/6 females, the genetic background of our transgenic line [51]. Nevertheless, testing DMT in females using validated depression models will be essential, since accumulating evidence points to sex-dependent responses to serotonergic psychedelics. For instance, psilocin produces distinct, time-dependent changes in central amygdala reactivity and defensive behavior between males and females [82], while DOI and LSD modulate prepulse inhibition in a sex- and 5-HT2A receptor–dependent manner in 129S6/SvEv mice [83]. This consideration extends beyond preclinical work, as a recent scoping review of psychedelic trials for substance use disorders revealed a pervasive underrepresentation of women and a near absence of sex-disaggregated analyses [84]. Mechanistically, fluctuations in ovarian hormones regulate serotonergic signaling, including 5-HT2A receptor expression, and intersect with neuroplasticity pathways such as BDNF/TrkB and mTOR. These interactions suggest that hormonal state and cycle phase could modulate both the magnitude and persistence of psychedelic effects [85]. Future studies should therefore, when possible, adopt sex-balanced designs across both preclinical and clinical settings. Incorporating these variables will be crucial not only for improving translational validity but also for ensuring that the emerging therapeutic use of psychedelics reflects the full spectrum of biological and experiential diversity across sexes.

In conclusion, our study provides preclinical evidence that a single dose of DMT can robustly reverse depression-like behaviors induced by chronic stress and enhance adult hippocampal neurogenesis. These effects persist under isoflurane anaesthesia and are stronger than those achieved by chronic fluoxetine. Together, our findings support the development of DMT as a rapid-acting antidepressant targeting circuit-level restoration. Given its broad impact on both affective and cognitive domains, we view DMT as a promising candidate for treating depression, particularly in individuals who do not respond to traditional monoaminergic therapies.

However, important questions remain. Future studies should investigate the dose-response relationship of DMT to better define its therapeutic window and optimize translational relevance, particularly in light of prior reports showing variable efficacy across dosing regimens in rodents. To better inform translational strategies, the pharmacokinetic and pharmacodynamic properties of DMT across species should also be explored. In parallel, efforts to delineate the precise neural circuits and receptor pathways mediating DMT’s effects, through pharmacological or circuit-level manipulations, will be essential to establish causal mechanisms. Longitudinal designs are also needed to assess the durability of therapeutic effects following single or repeated dosing. Moreover, as all experiments were conducted in male mice, validation in female subjects will be crucial for assessing generalizability and identifying potential sex-specific responses to DMT administration. Addressing these questions will be critical for advancing DMT-based strategies toward clinically viable, fast-acting interventions.

## Supplementary information

Supplementary Figure 1

Supplementary Figure 2

Supplementary Figure 3

Supplementary Table 1

## Data Availability

All original contributions presented in the study are included in the article and supplementary material, further inquiries can be directed to the corresponding authors.
