# Supplementary material for: Single-dose DMT reverses anhedonia and cognitive deficits via restoration of neurogenesis in a stress-induced depression model
Source: Transl Psychiatry. 2026 Jan 29;16:101. doi: 10.1038/s41398-026-03852-7 (PMC12923610; doi:10.1038/s41398-026-03852-7)
Supplement: Supplementary file 1 — Supplementary Figure 1 [file 41398_2026_3852_MOESM1_ESM.docx]

**Supplementary figure 1**

**
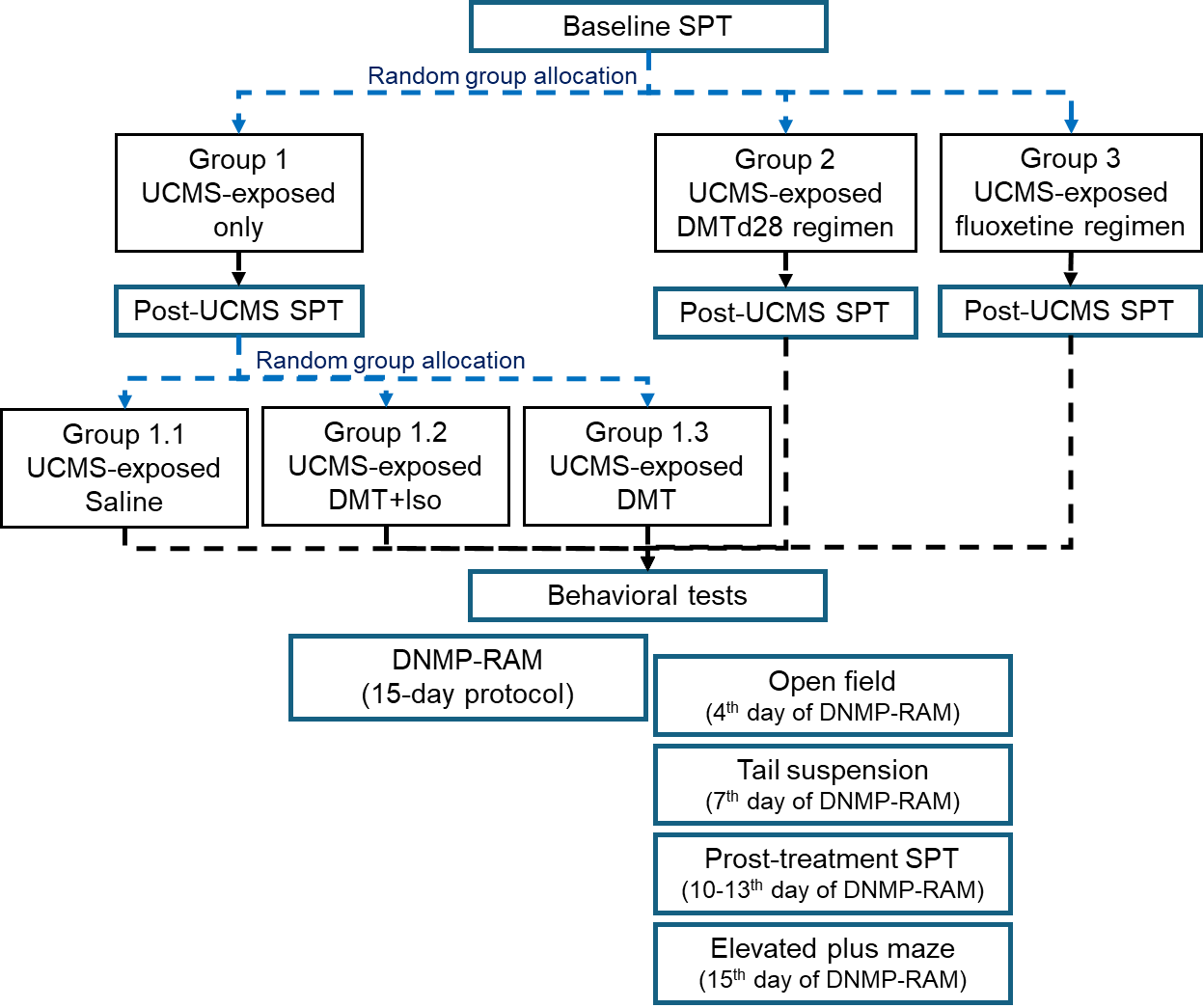
**

**Supplementary Figure 1. Experimental design and behavioral testing timeline.** The illustration represents the group allocation, experimental workflow and timeline for behavioral assays conducted in the study. Naïve non-UCMS animals underwent the same behavioral testing and are not represented in the flowchart. All animals underwent a baseline sucrose preference test (SPT) before exposure to UCMS. Mice were then assigned to one of three initial groups: UCMS-exposed only (Group 1), UCMS-exposed with DMT administration on day 28 (Group 2), or UCMS-exposed with fluoxetine treatment (Group 3). Following the UCMS period, post-UCMS SPT was performed prior to treatment administration. Next, Group 1 animals were further randomized to receive a single dose of saline (Group 1.1), DMT under isoflurane anesthesia (Group 1.2), or DMT alone (Group 1.3). All animals were subjected to a 15-day delayed non-match to position radial arm maze (DNMP-RAM) protocol. Other behavioral tests were performed simultaneously and included: open field test, tail suspension test (TST), post-treatment sucrose preference test, and elevated plus maze. This design enabled assessment of both cognitive and affective phenotypes across multiple domains.
