# Supplementary material for: Single-dose DMT reverses anhedonia and cognitive deficits via restoration of neurogenesis in a stress-induced depression model
Source: Transl Psychiatry. 2026 Jan 29;16:101. doi: 10.1038/s41398-026-03852-7 (PMC12923610; doi:10.1038/s41398-026-03852-7)
Supplement: Supplementary file 2 — Supplementary Figure 2 [file 41398_2026_3852_MOESM2_ESM.docx]

**Supplementary figure 2**

**
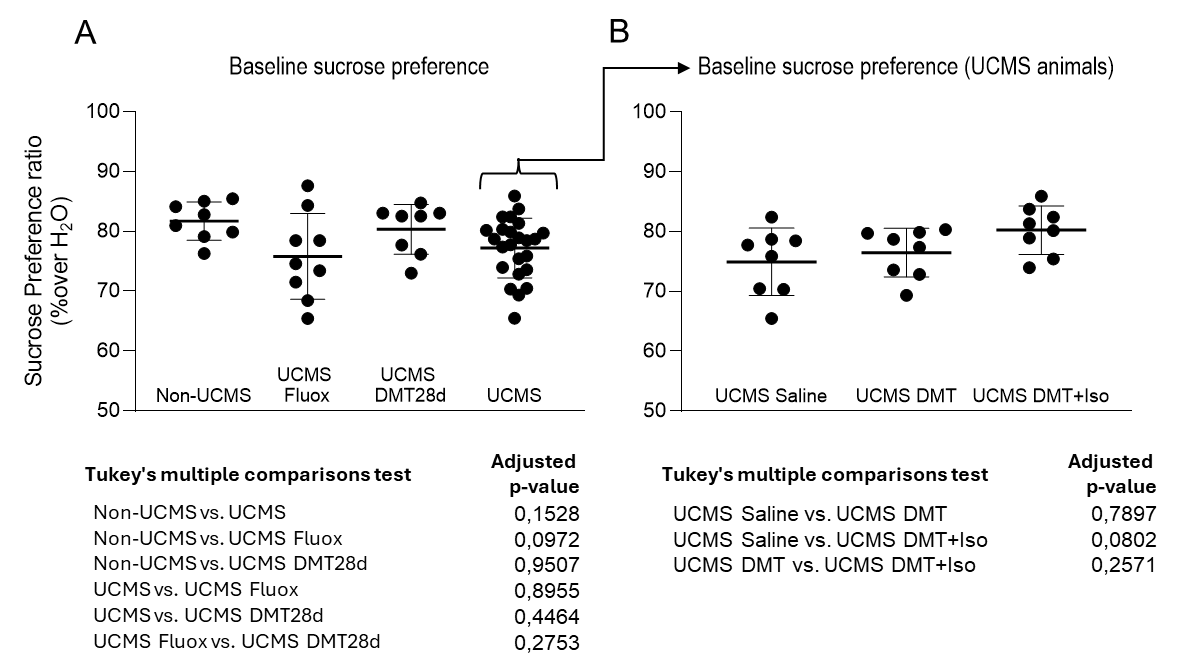
**

**Supplementary Figure 2. Baseline sucrose preference was balanced across experimental groups prior to treatment allocation.** (A) Mean ± SD of sucrose preference measured during baseline testing for animals assigned to Non-UCMS (n = 8), UCMS Fluoxetine (n = 9), UCMS DMT-d28 (n = 8), and UCMS-exposed (n = 24) groups. No significant differences in group means were found, as determined by one-way ANOVA followed by Tukey’s multiple comparisons test (see table). Additionally, there were no significant differences in standard deviation across groups, as assessed by Brown-Forsythe and Bartlett’s tests, confirming that variance was comparable across conditions prior to stress exposure.(B) Baseline sucrose preference scores for UCMS-exposed animals, stratified by their future treatment assignment (Saline, DMT, or DMT+Iso; n = 8 per group). No significant differences were detected in mean sucrose preference or variability between groups (similar one-way ANOVA and variance tests, see table), indicating that baseline hedonic behavior was evenly distributed before treatment randomization. Animals with baseline sucrose preference scores below 60% were excluded from all analyses as potential non-responders to the test, while animals with scores above 75% after UCMS were excluded as UCMS-resistant (i.e., stress-insensitive). Data shown include only animals meeting inclusion criteria (see Methods).
