# Supplementary material for: Single-dose DMT reverses anhedonia and cognitive deficits via restoration of neurogenesis in a stress-induced depression model
Source: Transl Psychiatry. 2026 Jan 29;16:101. doi: 10.1038/s41398-026-03852-7 (PMC12923610; doi:10.1038/s41398-026-03852-7)
Supplement: Supplementary file 3 — Supplementary Figure 3 [file 41398_2026_3852_MOESM3_ESM.docx]

**Supplementary figure 3**

**
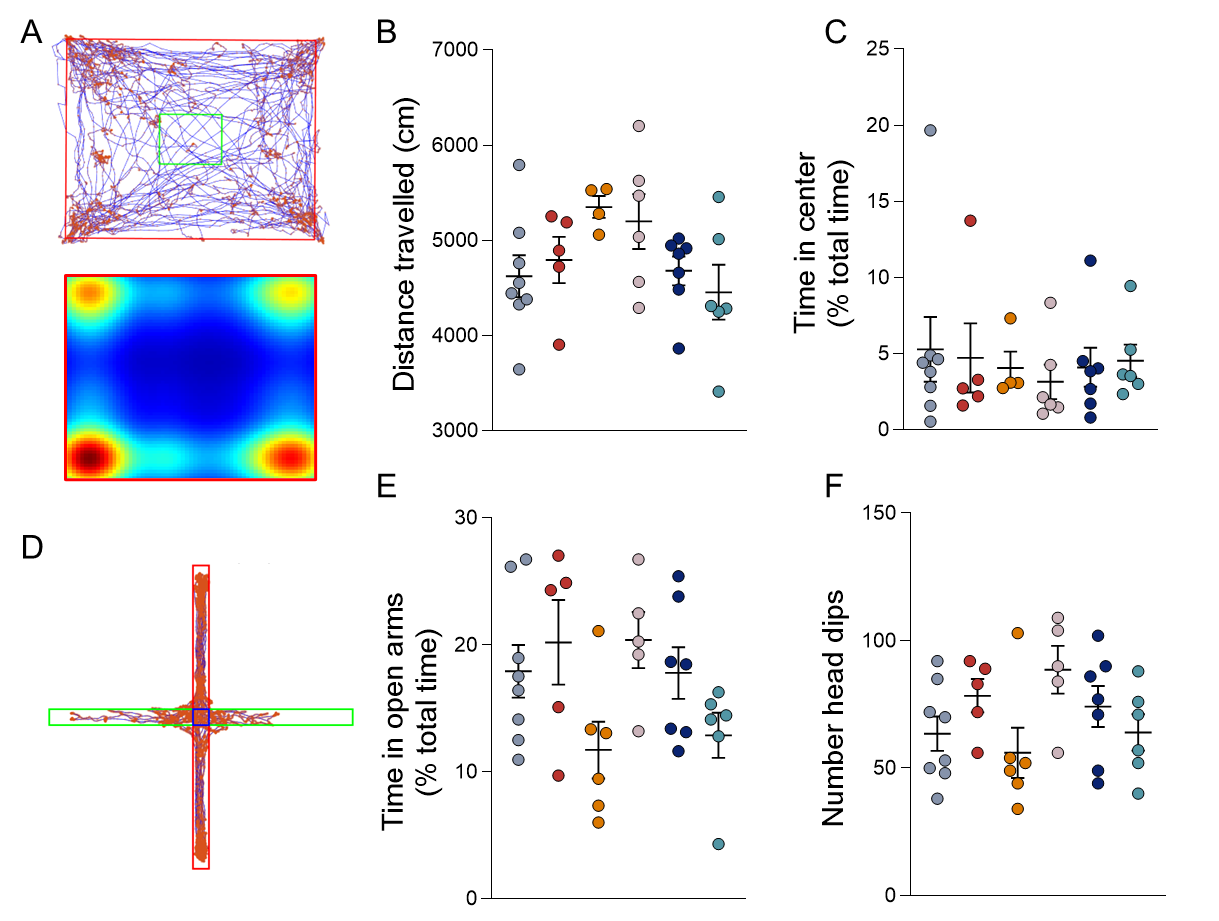
**

**Supplementary Figure 3. Anxiety-like phenotypes were assessed by open field and elevated plus maze tests. (A)** Representative illustration of movement tracking paths (top panel) and occupancy density plots (bottom panel) from the open field test of a representative mouse from the UCMS DMT group. **(B)** Quantification of total distance traveled (cm) in the open field test. **(C)** Percentage of time spent in the center area relative to total test duration in the open field test. **(D)** Representative illustration of movement tracking paths in the elevated plus maze from a representative mouse of the UCMS DMT group. **(E)** Percentage of total test time spent in the open arms of the elevated plus maze. **(F)** Number of head dips as an indicator of exploratory behavior in the elevated plus maze. All data are presented as individual values with mean ± SEM. Statistical analyses were conducted using one-way ANOVA followed by Tukey's post-hoc test. Sample sizes for open field test: Non-UCMS (n=8), UCMS Saline (n=5), UCMS Fluox (n=4), UCMS DMT (n=6), UCMS DMT+ISO (n=7), UCMS DMTd28 (n=6). Sample sizes for elevated plus maze test: Non-UCMS (n=8), UCMS Saline (n=5), UCMS Fluox (n=4), UCMS DMT (n=6), UCMS DMT+ISO (n=7), UCMS DMTd28 (n=6).
