# Supplementary material for: Single-dose DMT reverses anhedonia and cognitive deficits via restoration of neurogenesis in a stress-induced depression model
Source: Transl Psychiatry. 2026 Jan 29;16:101. doi: 10.1038/s41398-026-03852-7 (PMC12923610; doi:10.1038/s41398-026-03852-7)
Supplement: Supplementary file 4 — Supplementary Table 1 [file 41398_2026_3852_MOESM4_ESM.docx]

**Supplementary table 1**

|  | Monday | Tuesday |  | Wednesday | Thursday | Friday | Saturday | Sunday |
| --- | --- | --- | --- | --- | --- | --- | --- | --- |
| 1st week | BW; CS; Hot air jet | Shaking |  | Confinement | Rat feces | Confinement | Confinement | Tilted cage |
| 2nd week | BW; CS  Confinement | Shaking |  | Rat feces | Confinement | Shaking | Rat feces | Confinement |
| 3rd  week | BW; CS  Confinement | Rat Feces |  | Confinement | Hot air jet | Rat feces | Confinement | Shaking |
| 4th week | BW; CS  Rat feces | Confinement |  | Rat feces | Shaking | Confinement | Shaking | Hot air Jet |
| 5th week | BW; CS  Confinement | Tilted cage |  | Hot air jet | Confinement | Rat feces | Inverted Light cycle | Rat feces |
| 6th week | BW; CS  Hot air Jet | Shaking |  | Confinement | Tilted Cage | Nighttime illumination | Confinement | Hot air jet |
| 7th week | BW; CS  Confinement | Rat feces |  | Confinement | Hot air jet | Shaking | Confinement | Nighttime illumination |
| 8th week | BW; CS  Shaking | Confinement |  | Rat feces | Shaking | Confinement | Confinement | Shaking |
| 9th week | BW; CS |  |  |  |  |  |  |  |

**Supplementary Table 1:** Chronogram for the UCMS protocol. BW: Body Weight assessment; CS: Coat Score assessment.
